# Co-transfection of dendritic cells with AFP and IL-2 genes enhances the induction of tumor antigen-specific antitumor immunity

**DOI:** 10.3892/etm.2012.635

**Published:** 2012-07-06

**Authors:** JING-YUE YANG, XIAO LI, LI GAO, ZENG-HUI TENG, WEN-CHAO LIU

**Affiliations:** 1 Department of Clinical Oncology, State Key Discipline of Cell Biology, Xijing Hospital;; 2 Departments of Hepatobiliary Surgery and; 3 Dermatology, Xijing Hospital;; 4 Department of Pharmacology, Faculty of Preclinical Medicine, Fourth Military Medical University, Xian, Shaanxi 710032, P.R. China

**Keywords:** dendritic cells, cytotoxic T lymphocytes, immunotherapy, α-fetoprotein, IL-2

## Abstract

Dendritic cells (DCs) are highly efficient, specialized antigen-presenting cells and DCs transfected with tumor-related antigens are regarded as promising vaccines in cancer immunotherapy. The aim of the present study was to investigate whether DCs co-transfected with the α-fetoprotein (AFP) and human interleukin-2 (IL-2) genes were able to induce stronger therapeutic antitumor immunity in transfected DCs. In this study, DCs from hepatocellular carcinoma (HCC) patients were co-transfected with the IL-2 gene and/or the AFP gene. The reverse transcription-PCR (RT-PCR) data revealed that the DCs transfected with the adenovirus AdAFP/IL-2 expressed AFP and IL-2. The DCs co-transfected with IL-2 and AFP (AFP/IL-2-DCs) enhanced the cytotoxicities of cytotoxic T lymphocytes (CTLs) and increased the production of IL-2 and interferon-γ significantly compared with their AFP-DC, green fluorescent protein (GFP)-DC, DC or phosphate-buffered saline (PBS) counterparts. *In vivo* data suggested that immunization with AFP-DCs enhances antigen-specific antitumor efficacy more potently than immunization with IL-2-DCs or AFP-DCs. These findings provide a potential strategy to improve the efficacy of DC-based tumor vaccines.

## Introduction

An effective cancer immunotherapy approach requires the activation of host T cells capable of recognizing a tumor target antigen and T-cell activation requires the appropriate antigen presentation by antigen-presenting cells. Dendritic cells (DCs) are the most potent professional antigen-presenting cells, with an excellent ability to interact with T cells and initiate their responses (1,2). The antigen-presenting ability of DCs makes them attractive vehicles for the delivery of therapeutic cancer vaccines (3,4). Numerous studies have focused on finding a feasible and effective DC-based vaccine, including pulsing DCs with tumor antigen peptide or protein, transducing genes encoding tumor antigen into DCs and fusing tumor cells with DCs (5–8). Among these, the genetic modification of DCs to express tumor antigens has been documented to be efficient for inducing antitumor immunity. In our previous study (5), we used α-fetoprotein (AFP) gene-modified DCs (AFP-DCs) to explore the potential of a DC-based tumor vaccine against hepatocellular carcinoma (HCC). Despite the induction of significant cytotoxic T-lymphocyte (CTL) responses, the antitumor effect was limited. Other investigators have also reported that the results of DC-based therapeutic vaccines were far from encouraging in some animal models (9,10).

Interleukin-2 (IL-2) is a potent stimulator of lymphocyte proliferation and increases the activity of CTLs (11). The systemic administration of IL-2 *in vivo* has a broad range of immunologic effects, including the induction of specific T-helper cells, natural killer (NK) and lymphokine-activated killer (LAK) cells and autoantibody production in congenitally T cell-deficient mice (12,13), and the enhancement of the restoration of immune function in irradiated or cyclophosphamide-treated animals (14,15). IL-2 is capable of specifically enhancing alloimmune responses in normal or primed mice and is able to enhance the antitumor activity of adoptively transferred LAK cells or specifically immune T lymphocytes (16). Of note, IL-2 alone is able to mediate the regression of selected, established murine and human tumors by mechanisms that involve the stimulation of *in vivo* lymphoid proliferation in tissues and by the activation of host-derived T cells (17).

Based on these studies, we explored potential therapeutic regimens based on the combined AFP and IL-2 transfected DCs. Moreover, we aimed to determine whether IL-2 is able to potentiate the antitumor effects of AFP-transfected DCs *in vivo*. Thus, the study was designed to provide a rationale for the development of clinical trials in humans using DC-based vaccine strategies.

## Materials and methods

### Construction and production of recombinant adenovirus (Ad) encoding AFP and IL-2

The AdAFP and AdIL-2 vectors were constructed using the AdEasy system. Briefly, fragments of the AFP and IL-2 genes were cloned into the pTrack plasmid containing a CMV promoter. To generate the recombinant adenoviral plasmid, following linearization with *Pme*I, the pTrack-CMV-AFP or pTrack-CMV-IL-2 vectors were co-transformed into *E. coli* BJ5183 with supercoiled pAd-Easy-1. The resultant AdAFP and AdIL-2 plasmids were characterized by restriction endonuclease digestion. To generate the adenoviral vector particles, the *Pac*I-digested plasmids AdAFP and AdIL-2 were infected into 293 cells by calcium phosphate co-precipitation methods. The AdAFP and AdIL-2 virus particles were propagated and purified as described in the AdEasy system instructions. The control AdGFP vector containing a green fluorescent protein (GFP) gene under the control of a CMV promoter was plaque-purified and amplified in the 293 cells. After two cycles of purification by CsCl ultra-centrifugation, the adenoviral vector titers were determined as viral genome particle numbers.

### Preparation of DCs

DCs were prepared as described in a previous study (18). PBMCs from HLA-A2^+^ HCC patients were isolated by Ficoll-Hypaque (Sigma, St. Louis, MO, USA) density gradient separation. Cells were either used immediately or cryopreserved in agents containing 50% X-VIVO 15 medium, 40% fetal calf serum (FCS) and 10% dimethyl sulfoxide (DMSO; Sigma). The PMBCs were then cultured in serum-free X-VIVO 15 medium (Cambrex Bioscience, Walkersville, MD, USA) in 6-well plates at 37°C with 5% CO_2_. After 2 h, non-adherent cells were removed by gently washing with phosphate-buffered saline (PBS) solution. Adherent cells were replenished with 30 ml X-VIVO 15 medium containing 100 ng/ml granulocyte macrophage-colony stimulating factor (GM-CSF; Peprotech, Inc., Rocky Hill, NJ, USA) and 10 ng/ml interleukin-4 (IL-4; Peprotech) and incubated for 7 days at 37°C with 5% CO_2_.

### Generation and genetic modification of DCs

Ad vectors were purified by two rounds of CsCl density centrifugation, dialysed and stored at −70°C in 3% sucrose. Vector preparations were demonstrated to be free of replication-competent adenovirus. To assess the ability of the Ad vectors to transfer and express genes in DCs, the cells were infected with AdGFP for 2 h after 7 days of culture using a multiplicity of infection (MOI) of 50, 100 or 200. Two days later, GFP expression was quantified by flow cytometry. We observed a dose-dependent response to the adenoviral infections, with maximal staining (74%) at a MOI of ≥200. Therefore, a MOI of 200 was selected for the transfection of DCs with AdAFP and AdIL-2 in this study.

### Ad vector-mediated infection and AFP and IL-2 expression in DCs

The Ad-mediated genetic modification of DCs was carried out by incubating the DCs with AdGFP, AdAFP, AdIL-2 or AdAFP combined with AdIL-2 at a MOI of 200 for 2 h and then washing the cells twice with complete X-VIVO 15 medium. The above DC vaccines are referred to as GFP-DC, AFP-DC, IL-2-DC and IL-2/AFP-DC, respectively. The DCs were collected 24 h after genetic modification to evaluate the gene transfer efficacy. The genetically-modified DCs were subjected to reverse transcription-PCR (RT-PCR) analysis of AFP and/or IL-2 expression. The primers used were β-actin, forward: 5′-ACAATGAGCTGCGTGTGGCT-3′ and reverse 5′-TCTCCTTAATGTCACGCACGA-3′, with an expected PCR product of 344 bp. The specific forward primer for human IL-2 was 5′-AGCAAGCTTACCATGCAACTCCTGTC-3′ and reverse: 5′-GCGGATCCTTATGTTGAGATGATGC-3′, with a product size of 447 bp. The specific primers for human AFP were AFP1 (outer forward): 5′-CTCTTCCAGA AACTAGGAGAA-3′, AFP2 (outer reverse): 5′-CTCTTCAGC AAAGCAGACTT-3′, AFP3 (inner forward): 5′-GCTGACA TTATTATCGGACAC-3′, AFP4 (inner reverse): 5′-AGCCTC AAGTTGTTCCTCTGT-3′, with an expected product size of 282 bp.

### Detection of AFP and IL-2 release by enzyme-linked immunosorbent assay (ELISA)

The supernatants of the cells from the four groups (GFP-DC, AFP-DC, IL-2-DC and IL-2/AFP-DC) were collected. The AFP release in the supernatants was evaluated by ELISA using an AFP ELISA detection kit (CanAg Diagnostics, Gothenburg, Sweden) and the culture supernatants of the genetically-modified DCs were collected for detection of the production of IL-2 using an ELISA kit (R&D Systems, Minneapolis, MN, USA).

### Flow cytometric analysis

DCs (2×10^5^ cells) were washed and resuspended in PBS solution containing 0.02% sodium azide and 1% bovine serum albumin. The cells were incubated with various fluorochrome-conjugated monoclonal antibodies at 4°C for 20 min in the dark. Phytoerythrin (PE)-conjugated antibodies against CD83, HLA-DR and CD86 and fluorescein isothiocyanate (FITC)-conjugated antibodies against CD80 (BD Biosciences Pharmingen, San Diego, CA, USA) were used. The cells were then washed twice and fixed in PBS solution containing 1% formaldehyde. The phenotypes were analysed by flow cytometry using a FACScan analytical flow cytometer.

### CTL generation and interferon-γ (IFN-γ) release ELISA

The CD8^+^ T cells from the HLA-A2+ HCC patients were positively selected using an anti-CD8 isolation kit (Dynal Biotech). The cells were suspended in complete X-VIVO 15 medium. DCs transfected with Ad or treated with TNF-α were plated with the effector T cells at a ratio of 1:20 (1×10^5^ DCs: 2×10^6^ effectors) in a total volume of 2 ml in 24-well tissue culture plates and cultured for seven days at 37°C with 5% CO_2_. The effector T cells were then harvested, washed, counted and restimulated with newly infected and mature DCs. The effector cells were serially stimulated a total of three times. Five days after the third stimulation, IFN-γ release in the supernatants of the effector cells was evaluated by ELISA using an IFN-γ ELISA detection kit. In brief, the effector T cells were collected and incubated with the target cells (HepG2) at a 20:1 ratio in a total volume of 200 μl in 96-well plates for 24 h. The supernatants were harvested from the cultures and IFN-γ assays were performed according to the manufacturer’s instructions.

### Cytotoxicity assay

The CD8^+^ T cells were incubated with stimulators (GFP-DCs, AFP-DCs, IL-2-DCs and IL-2/AFP-DCs) at a ratio of 20:1 (2×10^6^ T cells vs. 1×10^5^ stimulators) in 24-well culture plates in X-VIVO 15 medium for 5 days at 37°C with 5% CO_2_. The cytotoxicity analysis was performed using a lactate dehydrogenase (LDH) release assay. Briefly, the effector T cells were collected and incubated with the target cells (HepG2, SMMC7721 and K562) at ratio of 40:1 in 96-microwell plates at 37°C with 5% CO_2_ for 4 h. The plates were then centrifuged for 5 min. Supernatants from each well (100 μl) were transferred to 96-flat bottom microwell plates and 100 μl of the LDH substrate mixture was added. After 15 min, the absorbance was measured at 570 nm with an ELISA reader (Denley Dragon MK2; Labsystems, Helsinki, Finland). The CTL-mediated cytotoxicity was calculated using the equation: Cytotoxicity=[1-(effector-target-effector)/target] x100%.

### Immunizations and tumor challenge

We subcutaneously injected 1×10^5^ IL-2/AFP-DCs or the same number of DCs, GFP-DCs, IL-2-DCs or AFP-DCs, or PBS into the right flank region of C57BL/6 mice. The same immunization was repeated once after 1 week. Tumor challenge was initiated by the subcutaneous injection of 2×10^5^ HepG2 cells into the rear leg of the immunized mice 1 week after the last immunization to evaluate the specificity of the antitumor immunity induced by the DC vaccines in the immunized mice. Tumor occurrence was observed every other day. The length and width of the tumor mass were measured with calipers every other day and the tumor size was expressed as 0.5 × (length + width).

## Results

### Ad-mediated IL-2 and/or AFP genetic modification of DCs

**Figure 1:**
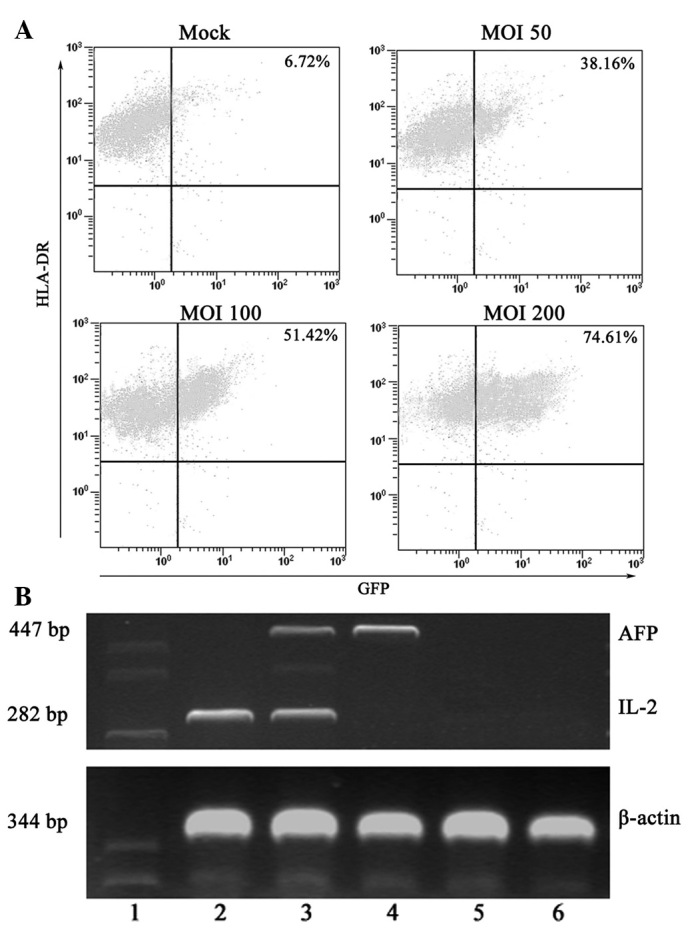
Transduction efficay of an Ad vector at various MOIs and surface marker expression of adenovirus-infected DCs. (A) Recombinant AdGFP was used to transduce day 7 immature DCs, which were then cultured for 48 h in the presence of GM-CSF and IL-4. On day 9, flow cytometric analysis of GFP expression by the Ad-GFP-DCs was carried out. Typically, >74% of cells were GFP^+^ at a MOI of 200. (B) IL-2 and/or AFP expression by gene-modified DCs is shown. PCR products of β-actin, IL-2 and AFP were visualized by electrophoresis in a 2% agarose gel containing 0.5 μg/ml ethidium bromide. Lane 1 Marker, lane 2 IL-2-DC, lane 3 IL-2/AFP-DC, lane 4 AFP-DC, lane 5 GFP-DC, lane 6 DC group. Ad, adenovirus; MOI, multiplicity of infection; DCs, dendritic cells; GFP, green fluorescent protein; GM-CSF, granulocyte macrophage-colony stimulating factor; IL-2, inter-leukin-2; AFP, α-fetoprotein; PCR, polymerase chain reaction.

The DCs were cultured *in vitro* for 7 days and then characterized using composite criteria of typical morphology. Flow cytometric analysis was performed 48 h after infection with AdGFP (at MOIs of 50–200) to determine the infection efficiency and the impact of Ad infection on the DC phenotypes. The DCs proved amenable to the *in vitro* Ad-mediated gene transfer and the efficiency was increased in a MOI-dependent manner. A MOI of 200 achieved transgene expression in 74.61% of cells (Fig. 1A). The GFP expression was stable and persisted while the DCs remained in culture.

Subsequently, we evaluated the efficacy of the Ad-transduced transfer of AFP and IL-2 genes into the DCs. IL-2 and/or AFP expression levels in the genetically-modified DCs were analysed by semiquantitative RT-PCR. As shown in Fig. 1B, DCs, GFP-DCs and IL-2-DCs did not express any detectable AFP, whereas AFP was detected in AFP-DCs and IL-2/AFP-DCs. IL-2 expression was detected in DCs transfected with or without various Ad, but the expression levels in DCs transfected with AdIL-2 alone or combined with AFP were significantly higher than in DCs, GFP-DCs and AFP-DCs. The results indicated that AFP and/or IL-2 genes were efficiently transfected. The culture supernatants of DCs following Ad transfection were collected and analysed for IL-2 production by ELISA.

**Figure 2:**
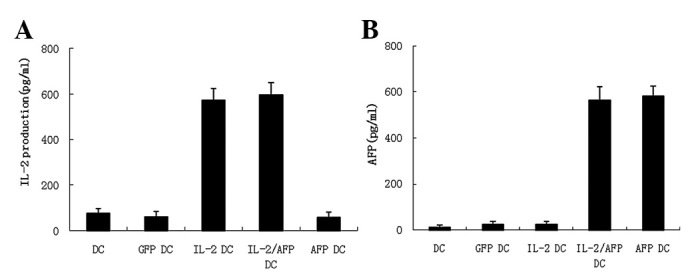
AFP and IL-2 release in the supernatants of infected DCs evaluated by ELISA. (A) IL-2 production by gene-modified DCs. The culture supernatants of DCs 24 h after Ad transfection were collected for measurement of IL-2 release by ELISA. Data are the mean ± SD of triplicate cultures. (B) In the supernatants of DCs transfected with IL-2/AFP (IL-2/AFP-DC) and AFP (AFP-DC), the levels of AFP were higher than in those transfected with GFP (GFP-DC) and IL-2 (IL-2-DC) (P<0.05); AFP release by DCs that were not transfected was at a very low level. DCs, dendritic cells; AFP, α-fetoprotein; IL-2, interleukin-2; GFP, green fluorescent protein; ELISA, enzyme-linked immunosorbent assay; Ad, adenovirus.

As shown in Fig. 2A, AFP release was detected in the supernatants of the DCs following transfection with AdIL-2/AFP and AdAFP. Although the levels of AFP were higher than in the supernatants from DCs transfected with AdIL-2 and AdGFP and untransfected DCs, they were nevertheless low levels and did not have any negative effect on the functions of DCs. In addition, the culture supernatants from DCs and genetically-modified DCs showed that IL-2 expression reached the highest levels of approximately 562.45 and 585.21 pg/ml at 24 h in the supernatants from the IL-2-DCs and IL-2/AFP-DCs, respectively, while <65 pg/ml IL-2 was detected in the supernatants from the untransfected DCs, GFP-DCs and AFP-DCs (Fig. 2B). The results indicate that low levels of IL-2 are secreted by DCs and that IL-2-genetically modified DCs are able to secrete IL-2 at relatively high levels.

### Gene transduction with the adenovirus vector did not impair DC function

**Figure 3:**
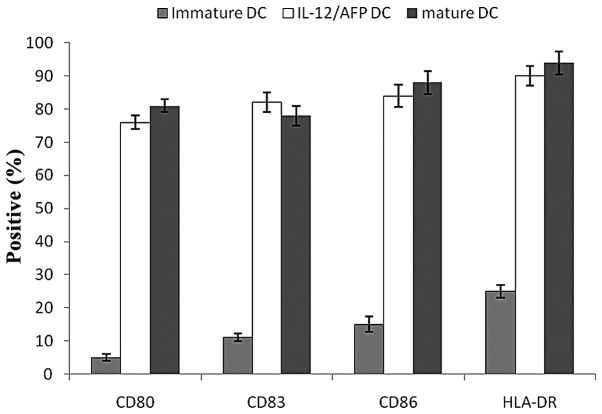
Cell surface markers of infected DCs measured by flow cytometry. Before DCs were infected with Ad, the expression levels of CD80, CD83, CD86 and HLA-DR were low, but following infection with Ad, the DCs expressed higher levels of CD80, CD83, CD86 and HLA-DR. Following infection with AdIL-2/AFP, the expression levels of CD80, CD83, CD86 and HLA-DR were 76.5, 82.1, 84.7 and 90.3%, respectively. The results indicate that the infected DCs exhibited a mature phenotypic change towards antigen presentation. DC, dendritic cells; IL-2, interleukin-2; AFP, α-fetoprotein.

DCs were assessed for cell surface phenotypes by flow cytometry prior to and following transfection. A greater degree of maturation was observed following transfection. This finding was characterized by an increased expression of the cell surface molecules CD83, CD80, CD86 and HLA-DR (Fig. 3). Genetically-modified DCs exhibited a phenotypic and functional change toward antigen presentation. Following transfection with Ad for 24 h, the levels of the cell surface molecules of the DCs were increased and the transfected DCs expressed higher levels of CD80, CD86, CD83 and HLA-DR compared with the immature DCs. Moreover, no significant differences in the expression levels of CD80, CD83, CD86 and HLA-DR were observed between the mature DCs and the infected DCs, indicating that gene transduction with the Ad vector did not alter the surface phenotypes of the DCs.

### More effective specific CTL response by DCs co-transfected with IL-2 and AFP

The cytotoxic activity of the DCs was assayed against HCC cells. Target cells comprised HepG2, SMMC7721 and K562 cells. The results indicated that IL-2/AFP-DCs specifically induced the highest CTL activity against AFP-expressing HepG2 cells. Moreover, the AFP-DCs also exhibited a more potent tumor-specific CTL response. CTLs were also induced by the IL-2-DC vaccine, but no significant CTL induction was observed by the GFP-DCs, DCs and T cells alone. Moreover, the CTLs specifically lysed the AFP-positive carcinoma cells, while the AFP-negative carcinoma cells were not lysed, indicating that the CTL response was antigen-specific (Fig. 5).

**Figure 4:**
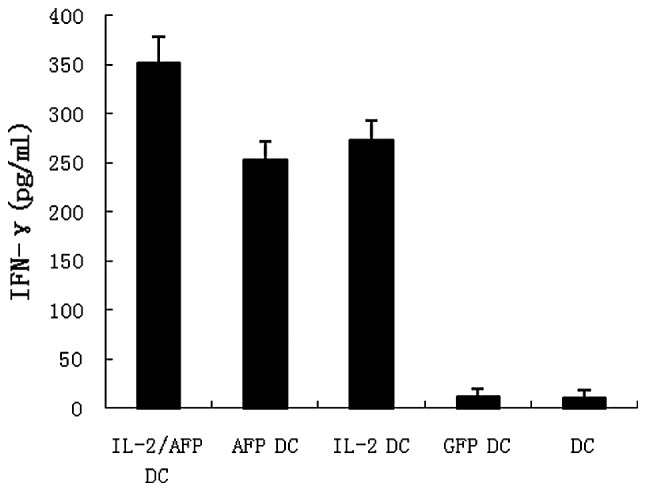
IFN-γ release in the supernatants of DCs evaluated by ELISA. IFN-γ release in the supernatants of CTLs co-cultured with HepG2 cells evaluated by ELISA. IFN-γ released by AFP/IL-2 DCs (347±26pg/ml) was higher than that released by AFP-DCs (243±18 pg/ml), IL-2-DCs (273±20 pg/ml), GFP-DCs (11±7pg/ml), DCs (9±8 pg/ml) and T cells. IFN-γ, interferon-γ; DCs, dendritic cells; ELISA, enzyme-linked immunosorbent assay; AFP, α-fetoprotein; IL-2, interleukin-2; GFP, green fluorescent protein.

**Figure 5:**
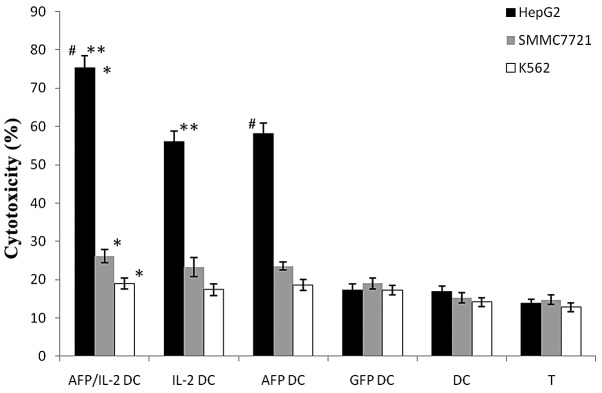
Cytotoxicity measured by LDH assay. The percentage of HepG2 cell-mediated lysis by IL-2/AFP-DCs (74.57±4.24%) was much higher than that of SMMC7721 and K562 cells, ^*^P= 0.0017; the percentage of lysed SMMC7721 cells was higher than that of lysed K562 cells. However, no significant differences among the lyses of the three target cells mediated by GFP-DCs, DCs or T cells were detected, P>0.05. For HepG2 cells, the percentage of lyses mediated by IL-2/AFP-DC were higher than those mediated by AFP-DCs (#P= 0.0421), IL-2-DCs (**P=0.0315), GFP-DCs, DCs and T cells alone, P<0.05. For SMMC7721 cells, the results were similar: the percentage of cell lyses mediated by IL-2/AFP-DC were higher than those mediated by AFP-DCs, IL-2-DCs, GFP-DCs, DCs and T cells alone, P<0.05. Lyses of K562 cells by CTLs, GFP-DCs, DCs or T cells alone were not significantly different, P>0.05. LDH, lactate dehydrogenase; IL-2, interleukin-2; AFP, α-fetoprotein; DC, dendritic cell; T, T cell; GFP, green fluorescent protein.

AFP-specific CTLs were generated as described in Materials and methods. IFN-γ release in the supernatants of the effector cells was evaluated by ELISA. The results revealed that IL-2/AFP-DCs co-cultured with HepG2 exhibited higher levels of IFN-γ than the other groups (Fig. 4).

### More effective elicitation of protective antitumor immunity by immunization with DC co-transfected with IL-2 and AFP

**Figure 6:**
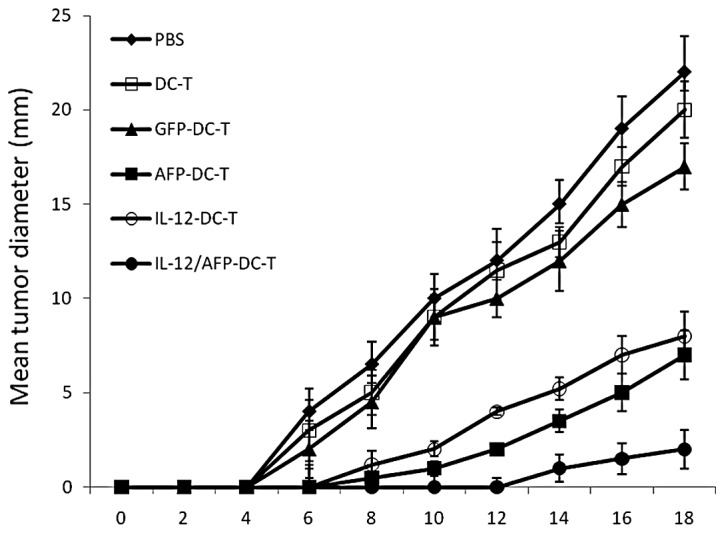
Induction of protective antitumor immunity by immunization with the IL-2/AFP DC vaccine. C57BL/6 mice were immunized subcutaneously with IL-2/AFP-DCs, AFP-DCs, IL-2-DCs, GFP-DCs or DCs 14 and 7 days prior to challenge with HepG2 cells. Tumor size was monitored with calipers every other day and calculated as the product of maximal perpendicular diameters. The results revealed that immunization with AFP-DCs or IL-2-DCs markedly inhibits tumor growth compared with immunization with GFP-DCs, DCs or PBS injection (P<0.01). Moreover, inhibition of melanoma growth was observed in mice following vaccination with IL-2/AFP-DC compared with mice vaccinated with other vaccines (P<0.01). IL-2, interleukin-2; AFP, α-fetoprotein; DC, dendritic cell; T, T cell; PBS, phosphate-buffered saline.

C57BL/6 mice were immunized subcutaneously twice with DCs, GFP-DCs, IL-2-DCs, AFP-DCs or AFP/IL-2-DCs. Seven days after the second immunization the mice were challenged with HepG2 cells. The results in Fig. 6 demonstrate that immunization with AFP-DCs or IL-2-DCs markedly inhibited the tumor growth compared with immunization with GFP-DCs or DCs or PBS injection (P<0.01). Additionally, inhibition of tumor growth was observed significantly in mice following vaccination with AFP/IL-2-DC compared with mice vaccinated with other vaccines (P<0.01). This result suggests that AFP/IL-2-DC is a potent vaccine that is able to induce specific antitumor immunity efficiently.

## Discussion

HCC is an aggressive disease and is the third highest cause of cancer death due to a lack of treatment options. Current efforts are now directed towards novel strategies for the treatment of HCC. One of the promising approaches is to design vaccines using DCs as the vehicle to deliver cancer antigens for an effective induction of T-cell antitumor immunity (19). To enhance the loading of DCs with tumor-associated antigen (TAA) *in vitro* and to further increase the efficacy of the DC vaccines, various techniques for the delivery of the priming antigen have been tested, including pulsing with peptide, protein or tumor cell lysates and transfection with viral vector-mediated TAA genes (20–25). Among these, gene transfer may be one of the most promising approaches as it may result in antigen processing naturally in the MHC class I and II pathways by DCs and stimulation of tumor-specific CTL and Th1 cells (26,27). Moreover, using an Ad vector to genetically modify DCs has been confirmed to be a good method due to its high efficacy and the minimum risk associated with insertional mutagenesis. In our experiment, the results revealed that the expression of Ad-GFP by infected DCs reached the highest level of 74.61%, which indicated efficient gene transfection. The co-stimulatory molecules CD80, CD86 and HLA-DR were upregulated significantly following Ad modification of the DCs compared with immature DCs, which demonstrated that the adenovirus transfection and gene expression did not affect on the DC maturation and antigen-presenting function.

AFP is a transcriptionally regulated protein expressed by most HCCs. Murine and human T-cell repertoires are reportedly able to recognize AFP despite being exposed to high plasma levels of this oncofetal protein during embryonic development (28–30). Therefore, AFP may be a target for the adjuvant immunotherapy of patients with HCC. In a previous study (5), we used AFP-DC as a cancer vaccine to evaluate the antitumor response. Despite the induction of specific CTL responses, the data demonstrated that the AFP-DCs elicited only limited antitumor immunity against HCC. Our results, together with those of other authors, suggest that the AFP-DC vaccine had to be improved to increase the antitumor efficacy.

IL-2 is important in the activation, differentiation and growth of hematopoietic cells, particularly T lymphocytes and NK cells. In several animal models, vaccination with such cytokine-transfected DCs induced rejection of the tumors and, in certain cases, protection against re-challenge with the parental tumor. In this study, we investigated the therapeutic effects of DCs co-transfected with AdIL-2 and AdAFP. We observed only small amounts of IL-2 in the supernatants of unmodified DCs, GFP-DCs or AFP-DCs which may be insufficient for the stimulation of T-cell proliferation and this may explain the failure of the AFP-DC vaccine to effectively control tumor growth compared with AFP/IL-2-DC, while a higher level of IL-2 production by the DCs was detected following IL-2 transfection. *In vitro* results suggested that AFP/IL-2-DCs enhance antigen-specific antitumor efficacy more potently than IL-2-DCs or AFP-DCs. In the animal experiment, AFP-DC and IL-2-DC vaccines inhibited the tumor growth significantly compared with the DC vaccine; however, the tumor inhibition by AFP/IL-2-DC vaccine was the most potent.

To conclude, the co-transfection of DCs with AFP and IL-2 genes may be further developed into a potential combination therapy strategy for adoptive cellular immunotherapy. Genetically-modified DCs offer a great opportunity for the immunotherapy of patients with HCC. This study therefore provides a promising strategy for a novel gene therapy for the treatment of HCC.

## Abbreviations:

Ad: adenovirus;
APCs: antigen-presenting cells;
CTLs: cytotoxic T lymphocytes;
DCs: dendritic cells;
GM-CSF: granulocyte macrophage colony stimulating factor;
IL-2: inter-leukin-2;
HCC: hepatocellular carcinoma;
IL-4: interleukin-4;
MOI: multiplicity of infection;
TAA: tumor-associated antigen
